# Metformin-loaded β-TCP/CTS/SBA-15 composite scaffolds promote alveolar bone regeneration in a rat model of periodontitis

**DOI:** 10.1007/s10856-021-06621-8

**Published:** 2021-12-04

**Authors:** Wanghan Xu, Wei Tan, Chan Li, Keke Wu, Xinyi Zeng, Liwei Xiao

**Affiliations:** 1 Department of Orthodontics, Medical Center of Stomatology, The Second Xiangya Hospital, Central South University, Changsha, 410011 Hunan PR China; 2 Department of Stomatology, Affiliated Xiaoshan Hospital, Hangzhou Normal University, Hangzhou, 311202 Zhejiang PR China; 3 Department of Spine Surgery, The Third Xiangya Hospital of Central South University, Changsha, 410011 Hunan PR China; 4 Department of Metabolism and Endocrinology, Hunan Provincial Key Laboratory for Metabolic Bone Diseases, National Clinical Research Center for Metabolic Diseases, the Second Xiangya Hospital, Central South University, Changsha, 410011 Hunan China; 5 Department of Cardiovascular Medicine, The Second Xiangya Hospital, Central South University, Changsha, Hunan 410011 PR China

## Abstract

Periodontitis is a progressive infectious inflammatory disease, which leads to alveolar bone resorption and loss of periodontal attachment. It is imperative for us to develop a therapeutic scaffold to repair the alveolar bone defect of periodontitis. In this study, we designed a new composite scaffold loading metformin (MET) by using the freeze-drying method, which was composed of β-tricalcium phosphate (β-TCP), chitosan (CTS) and the mesoporous silica (SBA-15). The scaffolds were expected to combine the excellent biocompatibility of CTS, the good bioactivity of β-TCP, and the anti-inflammatory properties of MET. The MET-loaded β-TCP/CTS/SBA-15 scaffolds showed improved cell adhesion, appropriate porosity and good biocompatibility in vitro. This MET composite scaffold was implanted in the alveolar bone defects area of rats with periodontitis. After 12 weeks, Micro-CT and histological analysis were performed to evaluate different degrees of healing and mineralization. Results showed that the MET-loaded β-TCP/CTS/SBA-15 scaffolds promoted alveolar bone regeneration in a rat model of periodontitis. To our knowledge, this is the first report that MET-loaded β-TCP/CTS/SBA-15 scaffolds have a positive effect on alveolar bone regeneration in periodontitis. Our findings might provide a new and promising strategy for repairing alveolar bone defects under the condition of periodontitis.

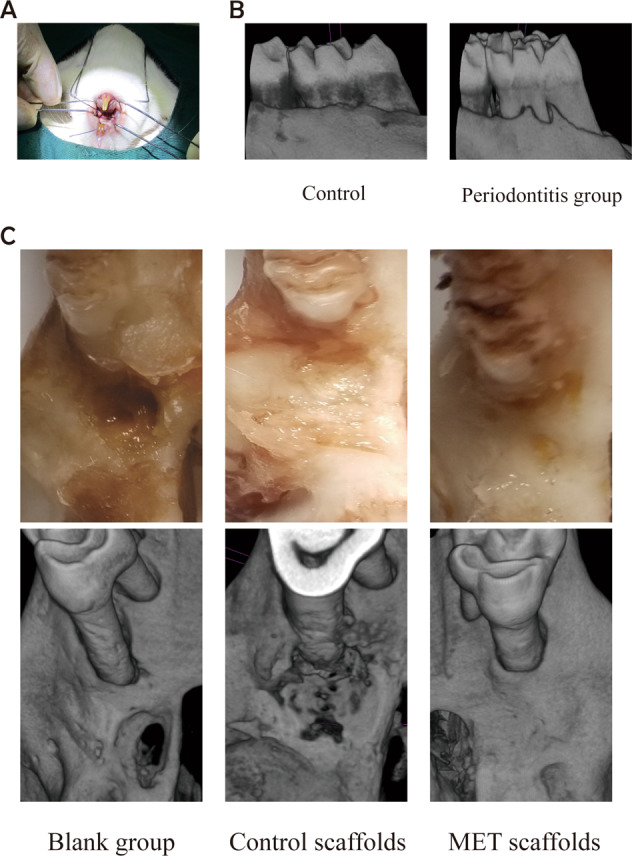

## Introduction

Periodontitis is a common chronic infectious disease with tissue destruction around teeth such as gingiva, periodontal ligament, and alveolar bone [1, 2]. Among them, alveolar bone defects were often unable to be healed naturally by the body’s repair mechanism. For oral treatment, alveolar bone resorption is the severe and irreversible damage associated with periodontitis. The complex oral inflammation environment poses a massive challenge for oral restoration and subsequent orthodontic treatment. At the same time, the loss of alveolar bone caused by periodontitis brings huge trouble to patients. To repair the residual alveolar bone defect in an inflammatory environment is a problem plaguing dentists, directly affecting the stability of subsequent dental implants. Bone grafting is one of the most effective methods to treat bone defects caused by periodontitis [3]. Although autogenous bone transplantation is extensively used to treat bone defects, it has disadvantages, including insufficient sources and related complications at the donor site [4, 5]. Furthermore, allogeneic bone is limited for clinical application due to rejection reaction and the risk of infection [6, 7]. With the development of biomaterial medicine, artificial bone scaffolds served as an alternative solution have shown promising potential to treat periodontal tissue bone defect. Many natural and synthetic materials such as collagen, glass ceramics, calcium sulfate, natural and artificial polymers are considered cell carriers and bone conduction materials [8]. Like the mineral phase of bone tissue, β-tricalcium phosphate (β-TCP) has appropriate biocompatibility, bioactivity, and osteoconductive ability. It also has unique features in biodegradation, solubility, and absorbance. Therefore, β-TCP or its composites are widely investigated as biomedical materials for bone defect repair and periodontal therapy [9, 10]. Chitosan (CTS) plays a crucial role in many biological activities, including antimicrobial and wound healing [11]. This material is applied to hemostasis, control of hypertension, drug delivery and has been extensively used as bone scaffold [12, 13]. More importantly, due to the positive effect on wound healing and anti-inflammation, CTS has received significant attention in dentistry. It can be applied in all fields of dentistry including preventive dentistry, conservative dentistry, endodontics, surgery, periodontology, prosthodontics and orthodontics. CTS demonstrated a considerable ability to reduce cariogenic bacteria, and the lack of cytotoxicity, which might enable its usage in the prevention of dental caries. The CTS-containing dentifrice had a higher protective potential against the demineralization of enamel, compared to the dentifrice without CTS [14]. As a representative silica-based mesoporous material, the mesoporous silica (SBA-15) is structured with uniform hexagonal pores and a tunable diameter of 5–15 nm. This mesoporous silica sieve has been attracting growing attention owing to its unique physico-chemical properties, such as high specific surface area, chemical inertness, narrow pore size distribution, sufficient active sites for grafting a variety of functional chemical groups, thermodynamic stability, and low cost. Based on these excellent characteristics, SBA-15 and its related hybrid materials have been broadly applied in selective adsorption, catalysis, drug delivery, imaging, and sensors [15]. Because of its good hydrothermal stability, drugs and growth factors of appropriate size are incorporated into the pores of the particles to form drug carrier complexes [16, 17].

Metformin (MET), an old antidiabetic drug, is widely used to treat type 2 diabetes by glycemic control [18]. Additionally, this drug has been shown to improve bone quality and decrease the risk of bone fractures in patients with diabetes. Increasingly studies have shown that MET can promote type I collagen synthesis and osteogenic differentiation in osteoblasts [19]. MET had an osteogenic effect by activating the AMP-activated kinase signal pathway to induce mesenchymal stem cells’ mineralization and osteogenic differentiation [20]. In vivo studies have also reported that MET had a positive effect against alveolar bone loss through osteoblasts differentiation in rats of periodontitis [21]. As discussed above, both in vitro and in vivo studies had demonstrated that MET might exhibit pro-osteogenic potential, which could be considered in treating bone loss disease.

In this study, we developed a new MET-loaded β-TCP/CTS/SBA-15 composite scaffold and then demonstrated that this synthesized scaffold could promote alveolar bone repair with periodontitis. We performed an animal model of alveolar bone defect with periodontitis and evaluated the role of this synthesized scaffold in alveolar bone repair. The potential effect of this MET-loaded composite scaffold on the alveolar bone repair with periodontitis has never been identified by previous investigators. This new composite scaffold might provide a new promising method for future bone regeneration therapy.

## Materials and methods

### Materials

All materials were purchased from Aladdin. CTS was in powder form (200–400 mPa.s), and MET came in a powder, crystals or chunks (purity: >97%). β-TCP was a white amorphous powder, odorless and tasteless (biomedical grade; particle size: 2–10 μm). SBA-15 was a nano-level white powder (particle size: 6–11 nm; relative crystallinity: ≥90%). Unless otherwise specified, all reagents were not required for further treatment.

### Preparation of MET-loaded β-TCP/CTS/SBA-15 scaffolds

The MET-loaded β-TCP/CTS/SBA-15 scaffolds were prepared via a freeze-drying method as published protocols [22]. The experimental scaffolds were prepared by mixing β-TCP, CTS, SBA-15, and MET at a mass ratio of 5:10:5:2. Briefly, MET and SBA-15 were thoroughly mixed in a centrifuge tube at low temperature for half an hour. The solution was fully mixed with CTS, β-TCP, and acetic acid. The mixture was poured into a mold and placed in a freeze-dryer cold trap. After pre-freezing at −20 °C for 2 h, porous scaffolds were obtained by lyophilizing at −80 °C for 24 h. Glutaraldehyde was dripped on the surface of the scaffold at low temperature for 1 h and rinsed with enzyme-free water twice. The above freeze-drying steps were repeated. The whole operation was carried out under strict sterile conditions, and the scaffold was stored at −80 °C before further experiments.

### Characterization of scaffolds

#### Metformin release from scaffolds

MET release from the scaffolds was measured. The scaffolds (*N* = 10) were placed in a centrifuge tube, and each centrifuge tube was added with 5 mL deionized water. 1 mL water of each centrifuge tube was collected for the measurement of MET release by Thermo spectrophotometer (NanoDrop 2000c) between 1 and 168 h at the specified timepoints. After each collection, 1 mL deionized water was supplemented into the centrifuge tube.

#### Mechanical behavior testing

The scaffold’s compressive strength and elastic modulus were determined using a universal mechanical testing machine (MTS, USA). Before conducting the mechanical test, the samples were ground to obtain a neat surface, followed by ultrasonic cleaning to remove debris and dry. The test samples were cylinders with a diameter of 6 mm and a height of 3 mm. The plate head was used, the speed was set at 0.5 mm/min, and pressure was slowly applied at 500 N. The experiment ended when the deformation exceeded 10%. The linear region of the stress-strain curve was used to determine the elastic modulus value. The elastic modulus of different groups was both of the mean value of 5 scaffolds.

#### Scanning electron microscopy (SEM)

The surface and internal morphology of the scaffold and the adhesion of bone marrow mesenchymal stem cells (BMSCs) to the scaffold were observed by scanning electron microscope (SEM, S-3400N). The control scaffolds used a gold-palladium coating of 10 nm, as previously reported [23]. The scaffolds with the cells were rinsed three times with phosphate buffer saline (PBS) and fixed in 2.5% glutaraldehyde and 1% osmium. Then the samples were dehydrated with ethanol and dried overnight. After sputtering gold was completed, the samples were investigated by SEM. By randomly selecting six fields of electron microscope photos, the maximum aperture of all scaffolds in each area of view was measured under the electron microscope.

#### Porosity measurement

The porosity of each sample was determined by the liquid replacement method. The porosity (ε%) was evaluated by subtracting the weight of scaffolds in wet (Ww) and dry (Wd) state. The scaffolds were immersed in anhydrous ethanol in the Ww measurement until they reached saturation and then weighed. The total porosity of the scaffold was determined using the following formula, where ρ represents the alcohol concentration (g/cm^3^), V represents the volume before immersion.

$$\varepsilon \% = \left( {{{{{{\mathrm{Ww}}}}}} - {{{{{\mathrm{Wd}}}}}}} \right)/\rho {{{{{\mathrm{V}}}}}} \times 100$$

#### FTIR

A Fourier transform infrared (FTIR) spectrophotometer was used to detect the spectra of components of the MET, β-TCP, CTS and SBA-15. Scaffolds in different groups were placed in the groove of the sample plate and pressed. The spectra of β-TCP/CTS/SBA-15 and MET/β-TCP/CTS/SBA-15 scaffolds were recorded in the wavelength range 500–4000 cm^−1^.

#### XRD

X-ray diffraction (XRD) was used to measure the sample’s phase composition over a diffraction angle (2θ) range of 5–80°, and the phase composition of the sample was analyzed using software according to the diffraction pattern.

### Animals

Three-month-old Sprague-Dawley male rats (300 ± 20 g) were included in the following experiments. All animal experiments were approved by the Ethics Committee of the Department of Experimental Zoology of Central South University and were under the guidelines for the ethical treatment of animals. The animals were given free access to food and water, and the feeding conditions were room temperature (25 ± 2 °C), humidity (60 ± 5%) under a 12-h light/12-h dark cycle. All rats were adapted to the environment for 7 days before the experiment.

### Harvest and culture of rat BMSCs and Alizarin Red staining

As previously reported, the whole bone marrow culture method was used to isolate rat BMSCs [24]. After removing the skin and soft tissues on the bone surface, both ends of the lower limbs in rats were cut off. The bone marrow washing fluid was immediately collected using bone marrow niches rinsing with PBS (Hyclone) under sterile conditions. Then the eluate was centrifuged at 1200 rpm for 5 min, and the supernatant was discarded. The pellet was resuspended in the complete culture medium supplemented with 10% fetal bovine serum (FBS, Gibco),1% penicillin-streptomycin (Gibco). The suspension was seeded in petri dishes and cultured in a saturated humidity incubator at 37 °C with 5% CO_2_. The third generation rat BMSCs were cultured in the osteogenic induction medium (DMEM supplemented with 10% FBS, 50 µM ascorbic acid, 10 mM β–glycerol‐phosphate and 10^−7^ mol/L dexamethasone (Sigma)) for 14 days. The medium was replaced every 2 or 3 days. On day 14 of induction, Alizarin Red staining evaluated calcium nodules on scaffolds and petri dishes.

### Biocompatibility

#### Cell viability assay

The cytotoxicity of the scaffolds was assessed using the cell counting kit-8 (CCK-8, Dojindo) assay. Briefly, BMSCs were seeded on the scaffolds in 96-well plates at a density of 2000 per well. According to the manufacturer’s protocol, on days 1, 3, 5, 7 of incubation, 110 μL solution containing a ratio of 10:1 from fresh medium and CCK-8 dye was added into each well and incubated in a CO_2_ incubator for 4 h. The absorbance was measured at 450 nm (OD_450nm_) using a microplate reader.

#### Live/dead cell staining

BMSCs were seeded on the scaffolds (5 × 10^5^ cells/scaffold) in 96-well plates. After 3 days of incubation at 37 °C and 5% CO_2_, the scaffolds were stained with a Calcein-AM/PI double staining kit (Solarbio). According to the instructions, the scaffolds were rinsed with PBS, stained with dye solution for 20 min in the dark. At the wavelength of 490 ± 10 nm, Calcein-AM detected yellow-green living cells, and propidium iodide (PI) detected red dead cells. The number and proportion of living and dead cells on the scaffold were analyzed by a confocal laser scanning microscope (LSM 780, AxioObserver, Zeiss).

### RT-PCR analysis

Rat BMSCs (5 × 10^5^) were seeded in a mixed liquid of MET/β-TCP/CTS/SBA-15 and β-TCP/CTS/SBA-15 scaffolds extract and osteogenic medium, and cultured in 6-well plates for 7 and 14 days in vitro. Cell pellets were obtained by removing the medium, digesting the samples with pancreatic enzymes and centrifuging the samples; the total RNA of the collected BMSCs were extracted using TRIzol reagent (TaKaRa, Japan). Reverse transcription was performed using the Revert Aid Reverse Transcriptase Kit (Thermo, USA), and RT-PCR was performed using SYBR^®^ Green PCR Master Mix (TaKaRa). All results were analyzed with StepOne software (Applied Biosystems, version 2.1) using the comparative CT method with GAPDH as the internal control. The expression levels of specific genes, including the osteogenesis-related genes Runx2, Col1a1, and BMP-2, were also analyzed. The primer sequences used in the PCR analysis are listed in Table 1.

**Table 1:** Sequences of the primers used in real-time qRT-PCR

Gene	Primer sequence				
	Forward		Reverse		
Runx2	ACCTCCAGGAAGCCTTTGAT		CCTGGTGGTGTCACTGAAT		
Col1a1	AACAAGGGAGGAGAGAGTGC		AGTCTCTTGCTTCCTCCCA		
BMP-2	CGGAAGCGTCTTAAGTCCAG		GCTAAGCTCAGTGGGGACAC		
GAPDH	GCAAGTTCAACGGCACAG		GCCAGTAGACTCCACGACAT		

### Alveolar bone regeneration in vivo

According to the previously published method, the periodontal model was induced by ligatures [25, 26]. Briefly, under sterile conditions, the rats were anesthetized with 1% pentobarbitone (0.4 mL/100 g) intraperitoneal injection. The maxillary area was exposed and placed a ligature (3-0) on the cervix of the first molar of each rat. The ligature was knotted at the mesial site and was gently pressed into the gingival sulcus below the gingival margin. It was necessary to peel off the gingival tissue in the cervix of the molar, causing mechanical damage to the gingival periodontal tissue and further aggravating the destruction of periodontal connective tissue and the loss of alveolar bone. Check the ligation every 2–3 days and re-ligate the molars again if it fell off. At the same time, the eating situation and gingival health status of rats were observed. After 4 weeks of gingival stimulation, dental calculus and plaque were accumulated at the ligated site, which stimulated inflammation and eventually developed into periodontitis.

The animals with periodontitis were randomly divided into three groups (*n* = 3) and treated as follows: (1) Experimental group: the alveolar bone defects were implanted with MET/β-TCP/CTS/SBA-15 scaffolds; (2) Control group: the alveolar bone defects were implanted with β-TCP/CTS/SBA-15 scaffolds; (3) Blank group: no material was implanted. After the periodontal model was established, the rats were anesthetized. The alveolar bone defects of 3 mm in diameter and depth were prepared with dental drills at the mesial site of maxillary first molars. In this process, continuous spraying of saline and intermittent and low-speed drilling were used to lower the temperature. Then the prepared scaffolds were immediately implanted into the bone defect, and the wound was sutured carefully. In the blank group, no material was implanted. Penicillin at 10,000 U/day was injected intraperitoneally for 3 days to prevent infection. The animals were sacrificed 12 weeks after the operation, and the alveolar bones were evaluated and tested.

### Radiographic and histological analysis

For the qualitative evaluation of the implanted scaffolds’ stability and bridging between the defect and the scaffold, the alveolar bone was scanned by microcomputer tomography (Micro-CT, Bruke Skyscan1176). At 12 weeks after surgery, alveolar bone samples were obtained, and Micro-CT examined the mineral formation in the defect area. The bone volume density (BV/TV), trabecular number (Tb.N), and trabecular bone thickness (Tb.Th) in the defect area were calculated by Micro-CT analysis software (CT Analyser). The samples were fixed with formalin, decalcified with 17% ethylenediaminetetraacetic acid solution for 4 weeks. Then the samples were dehydrated with graded ethanol and embedded in paraffin. 5 μm thick sections were cut from the middle of the scaffolds and stained with hematoxylin and eosin (H&E), and Masson.

### Statistical analysis

All data were expressed as the mean ± standard deviation (SD), and all experiments were performed at least three times. One-way analysis of variance (ANOVA) was performed to compare values using the Statistical Package for the Social Sciences (SPSS 23.0). A *P*-value of <0.05 was considered statistically significant.

## Results

### Cumulative drug release curve and mechanical behavior testing

**Fig. 1:**
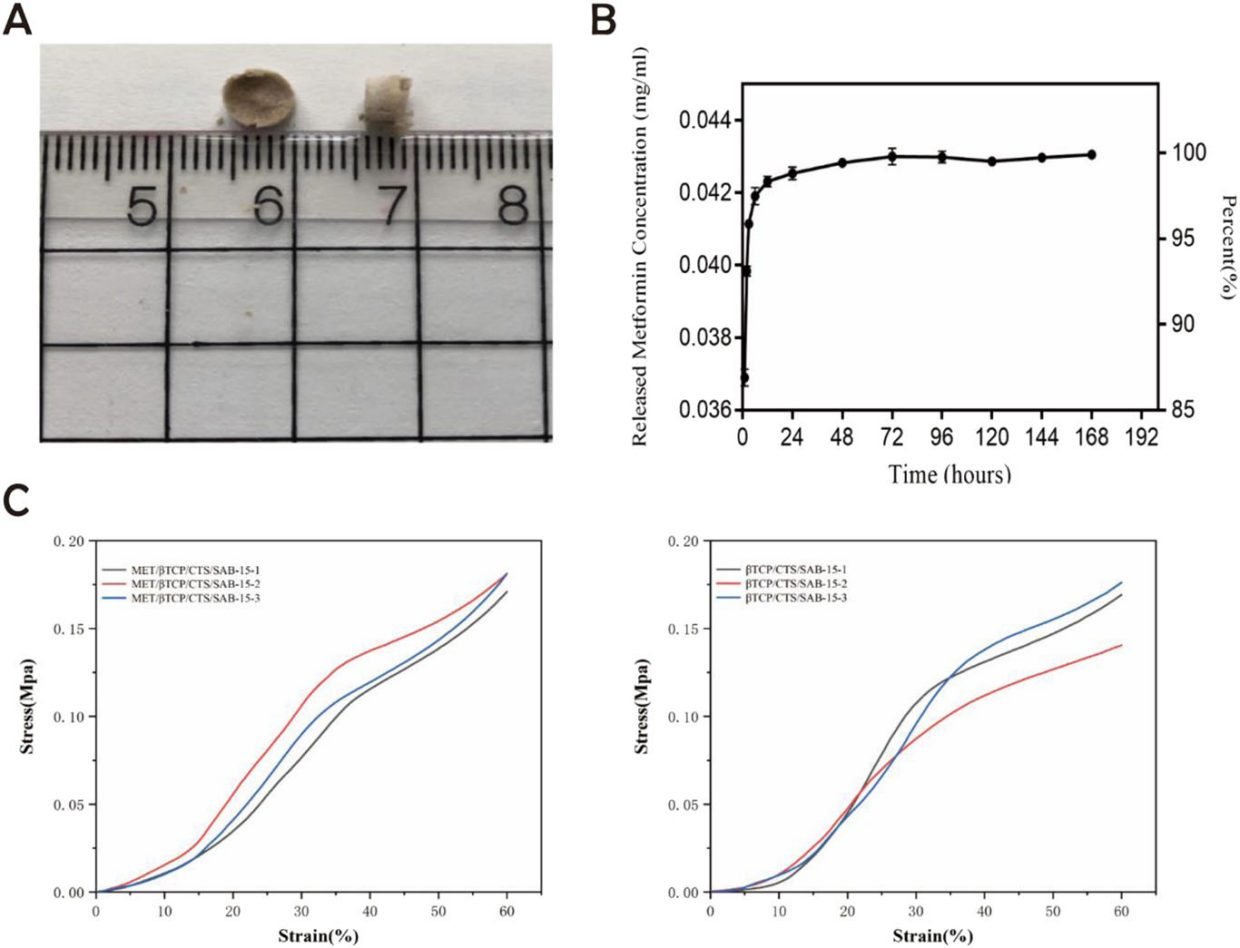
**A** Observation of scaffolds materials. **B** Metformin release from the MET/β-TCP/CTS/SBA-15 scaffolds. **C** Test the mechanical properties of the scaffolds in the MET/β-TCP/CTS/SBA-15 group and the β-TCP/CTS/SBA-15 group and plot the stress-strain curve

A cylindrical scaffold with a diameter of 6 mm and a length of 3 mm was prepared by the freeze-drying method (Fig. 1A). The results of the cumulative concentration of MET released from the scaffold were shown in Fig. 1B. The release process showed a fast release in the first 24 h, and the increase lasted for about 72 h. Then the MET concentration reached a plateau at 72–168 h. The mechanical performance test results showed (Fig. 1C) that the incorporation of MET did not affect the compressive strength and elastic modulus of the β-TCP/CTS/SBA-15 scaffold and MET/β-TCP/CTS/SBA-15 scaffold. (*P* > 0.05)

### Characterization of the MET/β-TCP/CTS/SBA-15 scaffolds

**Fig. 2:**
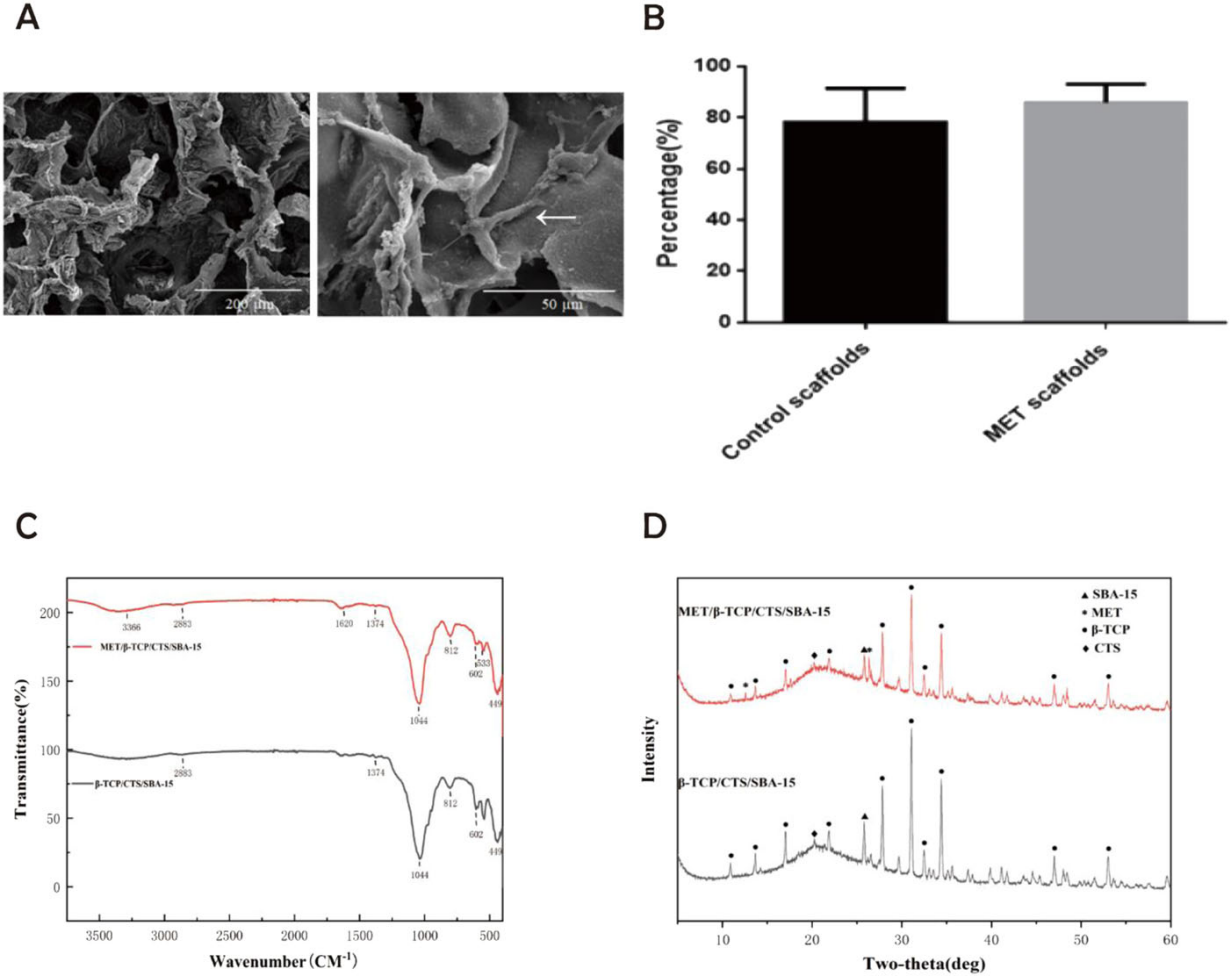
**A** SEM images of the MET/β-TCP/CTS/SBA-15 scaffolds seeded with BMSCs. The arrow indicated the pseudopodia. **B** The porosity of the MET/β-TCP/CTS/SBA-15 scaffolds and the β-TCP/CTS/SBA-15 scaffolds, respectively. **C** FTIR of MET/β-TCP/CTS/SBA-15 scaffolds and β-TCP/CTS/SBA-15 scaffolds. **D** XRD of MET/β-TCP/CTS/SBA-15 scaffolds and β-TCP/CTS/SBA-15 scaffolds

The internal structures of scaffolds were analyzed by SEM (Fig. 2A). The scaffolds had an apparent pore size with sufficient and interconnected pores, which provided a more suitable microenvironment for nutrient exchange and promoted the adhesion, proliferation of transplanted cells. BMSCs had good adhesion on the scaffold and were attached to the surface of the scaffold through pseudopodia. The porosity of the scaffolds was about 80% (Fig. 2B) and potentially meeting the requirements for tissue engineering applications as reported in the literature. The porosity of the scaffolds in the MET/β-TCP/CTS/SBA-15 group and the β-TCP/CTS/SBA-15 group was similar. The experimental test results showed that the incorporation of MET did not affect the porosity of the β-TCP/CTS/SBA-15 scaffold and MET/β-TCP/CTS/SBA-15 scaffold.

FTIR of the scaffolds was shown in Fig. 2C. The characteristic peaks of β-TCP could be seen at 602 cm^−1^, the characteristic peaks of CTS could be seen at 2883 and 1374 cm^−1^, the characteristic peaks of SBA-15 could be seen at 1044, 812, and 449 cm^−1^ and the peaks of MET could be seen at 3366, 1620, and 553 cm^−1^. The XRD test results were shown in Fig. 2D. The wave peaks corresponding to β-TCP, CTS, and SBA-15 could be seen in scaffolds in both groups. With the increase in the MET content in the MET/β-TCP/CTS/SBA-15 group samples, the size of the MET wave peaks increased. In contrast, the heights of the peaks corresponding to β-TCP, CTS, and SBA-15 decreased slightly, indicating that we successfully prepared the scaffold by combining the three components. With the addition of MET, the proportions of β-TCP, CTS, and SBA-15 gradually decreased, and the heights of their characteristic peaks decreased. The characteristic peaks of MET appeared in the MET/β-TCP/CTS/SBA-15 scaffold, indicating successful incorporation of MET into the scaffold.

### Characterization of rat BMSCs and Alizarin Red Staining

**Fig. 3:**
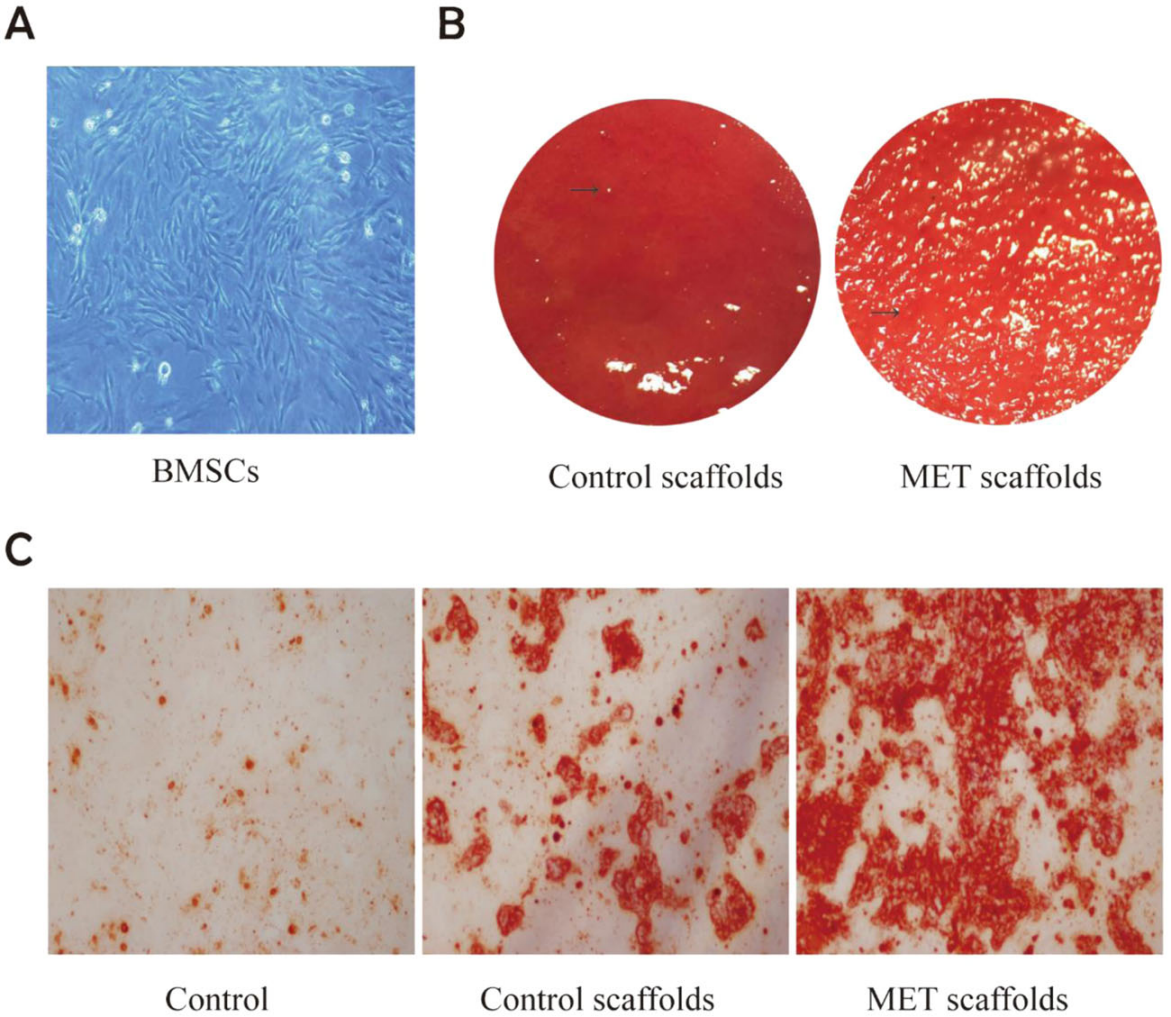
**A** Morphology of the third generation rat BMSCs. **B** The scaffolds in the β-TCP/CTS/SBA-15 group and the MET/β-TCP/CTS/SBA-15 group were cultured for 14 days after inoculation with rat BMSCs, stained with alizarin red. The arrow indicated the mineral nodules. **C** Incubate rat BMSCs with ordinary osteogenic induction medium, scaffold extract of β-TCP/CTS/SBA-15 group plus osteogenic medium, and MET/β-TCP/CTS/SBA-15 group scaffold extract plus osteogenic medium. Then stain BMSCs with alizarin red

The cells of passage 3 reached 70–80% confluence in a week of culture, and most cells showed a fibroblast-like shape and formed colonies (Fig. 1A). The osteoblast induction culture medium was incubated with the rat BMSCs seeded on the culture dish and the scaffold, and then stained with Alizarin Red 14 days later. The mineralized matrix synthesized by BMSCs covered the surface of the MET scaffold, which was thick and abundant. In contrast, in the control group, there were far fewer mineral nodules on the surface of the β-TCP/CTS/SBA-15 scaffold, and the scaffold surface was smoother (Fig. 3B).

The typical Alizarin Red stained image of BMSCs mineral synthesis was shown in Fig. 3C. The osteogenic induction medium, the osteogenic induction medium mixed with the control scaffold extract, and the osteogenic induction medium mixed with the MET scaffold extract were used to incubate BMSCs, respectively. After 14 days, orange-yellow calcium deposits in the cell dishes could be observed under the microscope, which confirmed that the BMSCs we extracted could be induced to differentiate into osteoblasts and had osteogenic potential. A large number of calcium deposits could be observed in the petri dishes of the experimental group, which had more calcium deposits than the β-TCP/CTS/SBA-15 scaffold group. Moreover, it could be observed that compared with the culture dishes in which stem cells were incubated only with ordinary osteogenic induction medium, the latter two had more abundant mineral deposits. It suggested that the scaffold material had particular osteoinductive potential.

### Biocompatibility

**Fig. 4:**
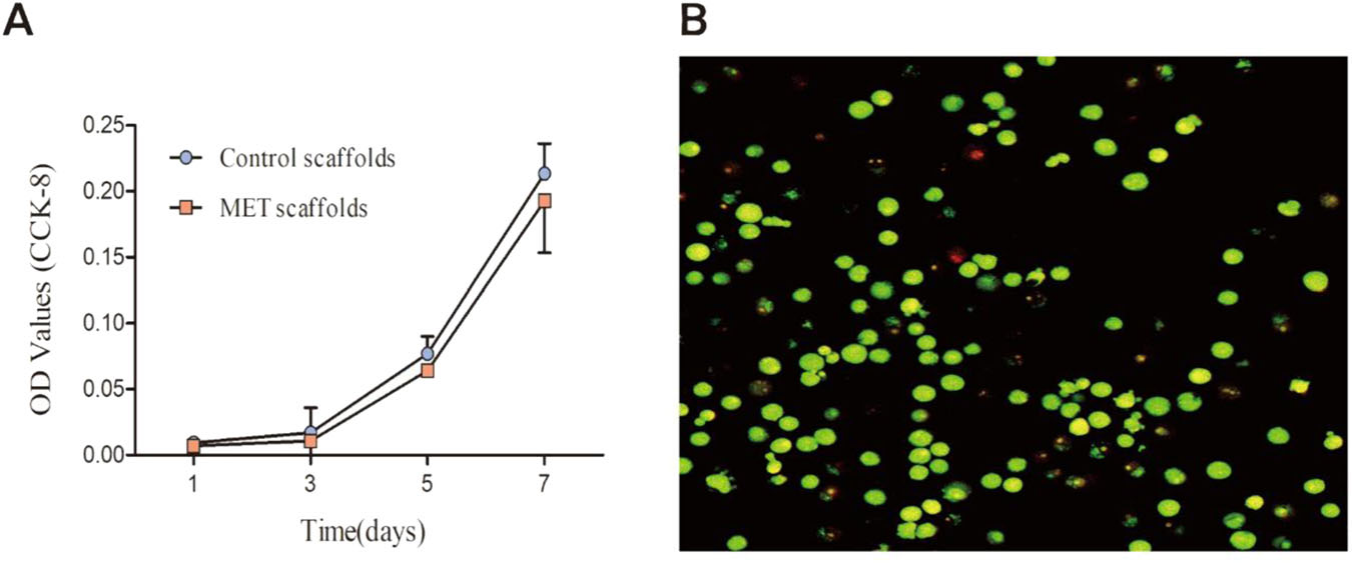
**A** The proliferation of BMSCs attached on scaffolds was confirmed by CCK-8 assay. **B** Representative live/dead images of BMSCs on MET/β-TCP/CTS/SBA-15 scaffolds, with live cells stained green and dead cells stained red

The cell viability of stem cells on the composite scaffold was assessed by CCK-8 assay and live/dead cell staining, respectively. Over time, the number of cells on the scaffolds increased during 7 days of incubation, consistent with the increase in absorbance (Fig. 4A). However, there was no statistical difference in cell growth rate between the two groups. This data proved that the incorporation of MET had no adverse effects on cell viability and proliferation. Results of the live/dead cell staining on the third day showed that a large number of live cells (green fluorescence) and a few dead cells (red fluorescence) on the MET/β-TCP/CTS/SBA-15 scaffolds could be observed under a confocal microscope (Fig. 4B). We found that cell immunofluorescence staining was consistent with the results of the CCK-8 assay. The results of these cytotoxicity tests indicated that the composite scaffolds had good biocompatibility.

### RT-PCR

**Fig. 5:**
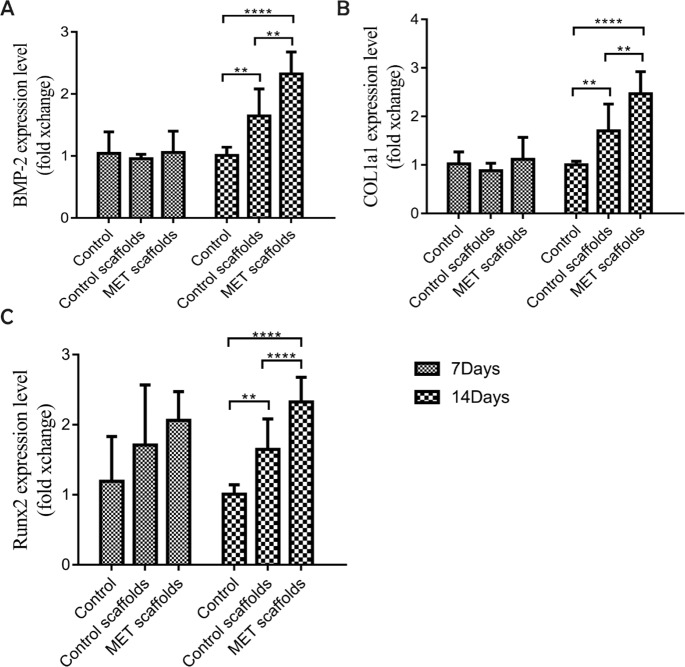
**A** Effects of the scaffolds on mRNA expression of the BMP-2 gene on days 7 and 14. **B** Effects of the scaffolds on mRNA expression of the COL1a1 gene on days 7 and 14. **C** Effects of the scaffolds on mRNA expression of the Runx2 gene on days 7 and 14. Symbol “**” shows the significant difference between groups (*P* < 0.05). Symbol “****” shows the significant difference between groups (*P* < 0.01)

As shown in Fig. 5, the RT-PCR results showed that the BMP-2, COL1a1, and Runx2 gene expression levels in BMSCs in the MET/β-TCP/CTS/SBA-15 group were higher than those in the β-TCP/CTS/SBA-15 group, with statistically significant differences between the two groups.

### Alveolar bone defect regeneration using MET/β-TCP/CTS/SBA-15 scaffold

**Fig. 6:**
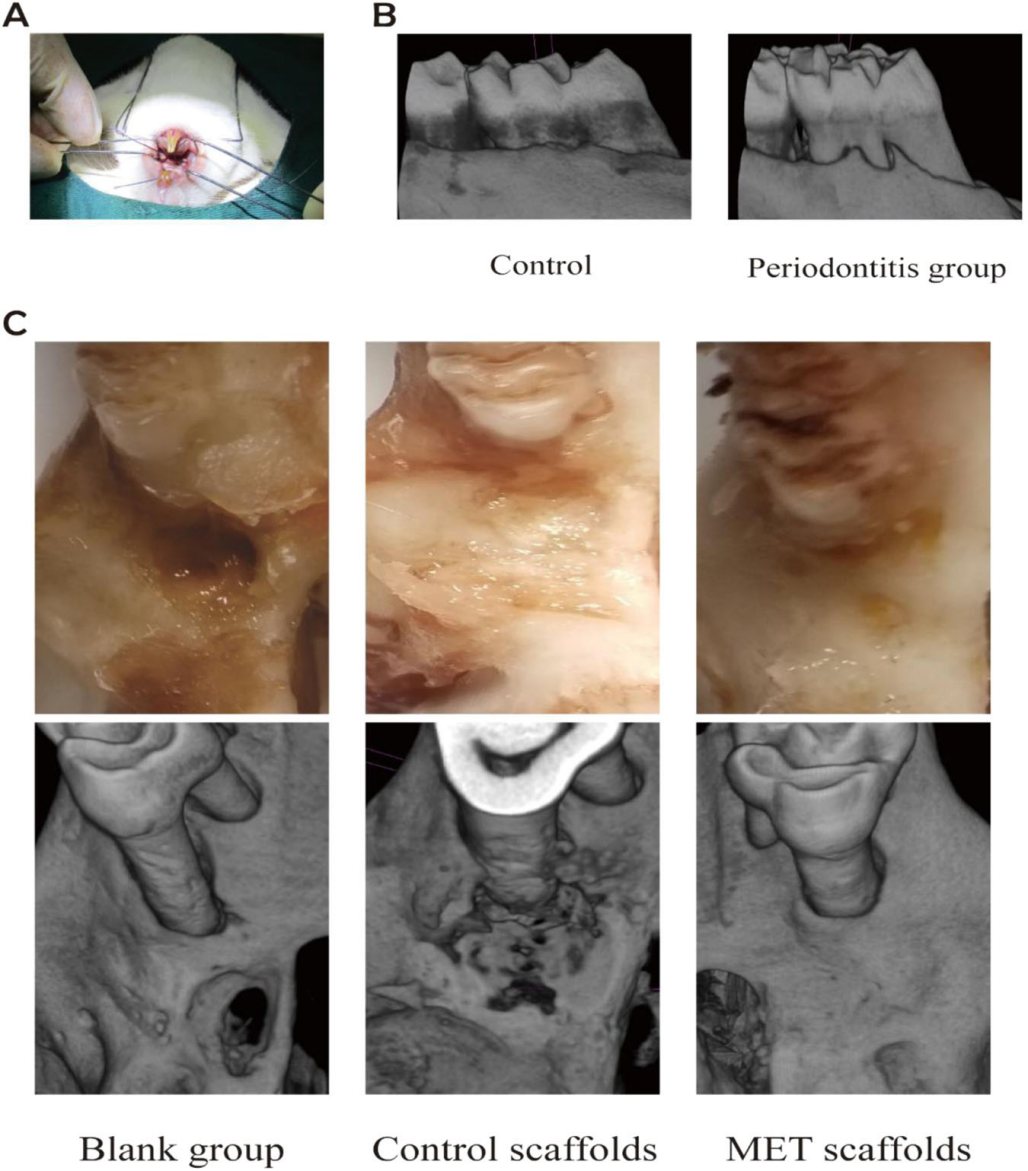
**A** The operation of the periodontal model. **B** Scan the periodontal tissues around the molars of the periodontitis group and normal healthy rats with Micro-CT. **C** Repair of alveolar bone defect in MET/β-TCP/CTS/SBA-15 group, β-TCP/CTS/SBA-15 group and blank group

In the rat model of periodontitis induced by ligation (Fig. 6A), red and swollen gums, bleeding on probing, and periodontal pockets could be observed. Micro-CT scan showed that compared with the control group, the alveolar bone of the rats in the periodontitis group was low and flat. Part of the alveolar bone was lost, root furcations were detectable (Fig. 6B), and the rat model of periodontitis was successfully established. The scaffolds of each group were implanted into the alveolar bone defect of Sprague-Dawley rats with periodontitis. At 12 weeks, the bone specimens were examined by Micro-CT. NRecon reconstructed the images to create 3D images of alveolar bone. Most of the bone defect in the MET/β-TCP/CTS/SBA-15 group had been repaired, while most of the bone defect in the β-TCP/CTS/SBA-15 group still existed and the bone defect repair was limited, and there was almost no bone defect repair in the blank group (Fig. 6C).

**Fig. 7:**
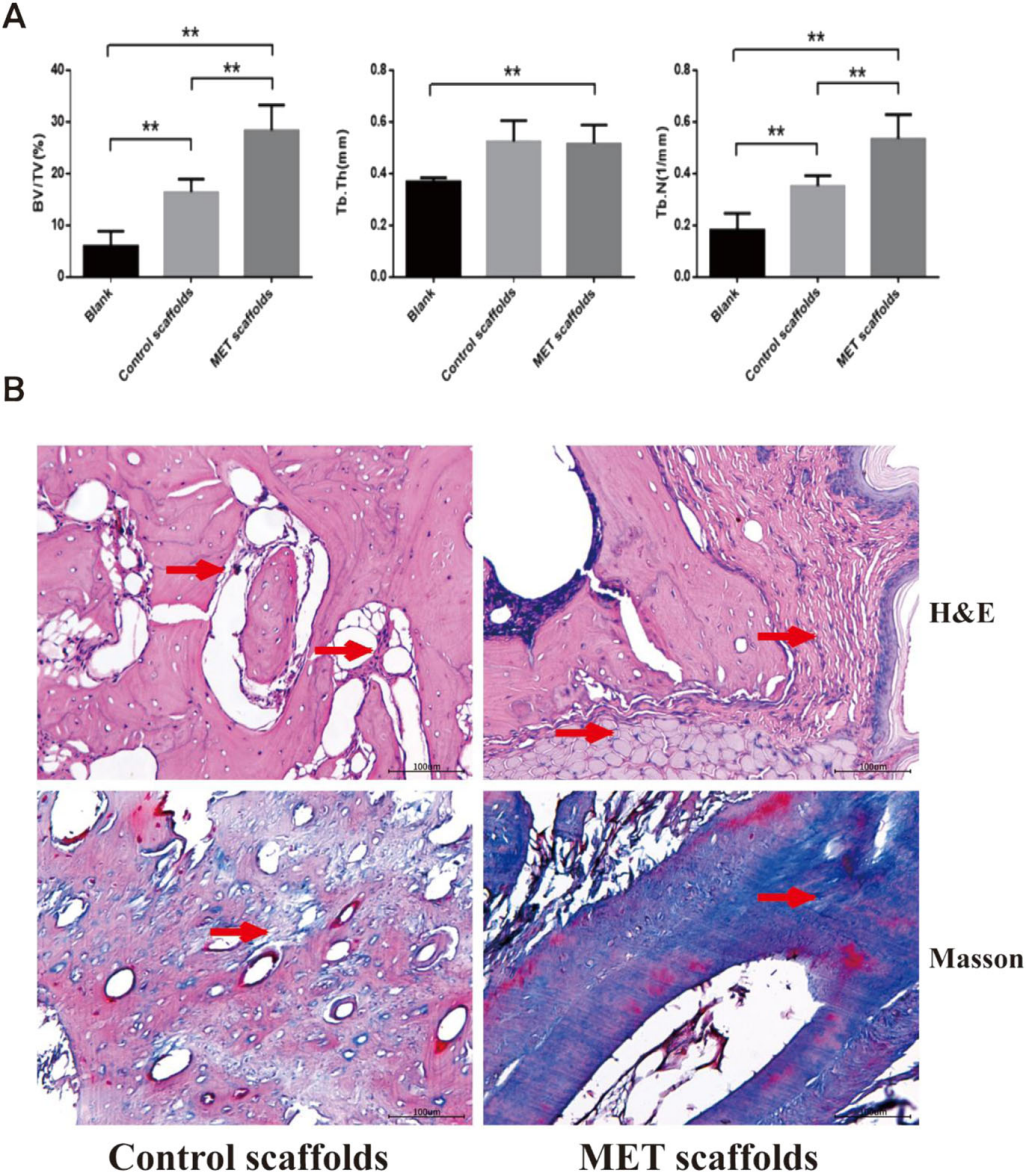
**A** Measurements of BV/TV, Tb.Th, and Tb.N of the alveolar bone. BV/TV trabecular bone volume fraction, Tb.N trabecular number, Tb.Th trabecular thickness. **B** Images of H&E staining, Masson staining of the alveolar bone. The red arrow indicated new bone formation. Symbol “**” shows the significant difference between groups (*P* < 0.05)

Also, the BV/TV and the Tb.N of the MET/β-TCP/CTS/SBA-15 group were better than those of other groups (Fig. 7A). Histological results further confirmed the Micro-CT results. H&E staining showed that a certain amount of new bone was formed at both the center and periphery of the bone defect in the MET/β-TCP/CTS/SBA-15 group, while a small amount of new bone was mainly formed around the junction of the β-TCP/CTS/SBA-15 scaffold and the bone defect. Masson staining results showed that the MET/β-TCP/CTS/SBA-15 group had abundant collagen fiber formation. Unlike the normal bone tissue, there was a little bone formation in the central area of the defect, and the arrangement of new bone was disordered in the β-TCP/CTS/SBA-15 group. (Fig. 7B).

## Discussion

As one of the two primary diseases of the oral cavity, periodontitis could cause periodontal tissue destruction, tooth looseness and loss, and gum inflammation. Alveolar bone destruction is its most crucial diagnostic feature. The treatment of periodontitis is a hot topic currently studied by scholars at home and abroad, and it is essential to find reasonable and effective drugs. Inhibition of bone resorption and promotion of bone regeneration is the key point in treating bone defects induced by periodontitis. MET is a commonly used antidiabetic drug. In vitro and in vivo studies had shown that MET stimulated mesenchymal stem cells to differentiate into osteoblasts and was used for tissue regeneration [27, 28]. MET at a 50 mg/kg concentration decreased bone loss, oxidative stress, and inflammatory response of ligature-induced periodontitis in rats [19]. Recent clinical evidence also indicated that locally delivered MET significantly improved chronic periodontitis radiological and clinical parameters. MET gel in the periodontal pockets of patients with chronic periodontitis could significantly reduce the probing depth (PD), increase clinical attachment level, and improve intrabony defects depth reduction in vertical bone [29, 30]. Systemic administration might cause unnecessary drug loading to other parts, causing undesirable side effects and significantly reducing the efficacy of drugs at the infected site. In contrast, the dose required for topical administration to produce a therapeutic effect at the target site was much lower [31, 32]. Therefore, composite scaffold materials were widely used for the repair of bone defects. In this study, we synthesized the new MET-loaded β-TCP/CTS/SBA-15 scaffolds by using the freeze-drying method, which played a role in limiting periodontitis and repairing alveolar bone defects. β-TCP and CTS were the matrix material of the scaffold. β-TCP mainly played a role in promoting bone healing. In addition to its anti-inflammatory and wound healing effects, CTS had the effect of adsorbing drugs, and it could slow-release MET. SBA-15 was mesoporous silica, which has the most vital drug absorption capacity and was the main component of the scaffold for drug absorption and sustained release. Our study is the first to report on the use of MET-containing scaffolds that repair alveolar bone defects under periodontitis.

The ideal scaffold material not only repairs bone defects and stabilizes the skeleton but also has non-toxicity and good biocompatibility. The use of a drug is a double-edged sword. Low-concentration drugs are non-toxic to all cells but may not be effective. The high concentration of the drug may be toxic to all cells, but the effect is significant. Therefore, we need to find a suitable concentration of MET, which can reduce drug’s toxicity to cells and ensure that the drug exerts a specific effect and promotes the osteogenic differentiation of stem cells. According to previous experimental reports, 50–500 μM MET effectively promoted stem cells proliferation and osteogenic differentiation [19]. Compared with untreated cells, stem cells treated with 250 and 500 μM MET significantly increased mineralization at these concentrations [28]. The drug release curve of the MET/β-TCP/CTS/SBA-15 scaffold indicated that the cycle of drug release was long, and the final concentration of MET could reach 0.043 mg/mL, which was within the above range. The experimental results showed that both the BMSCs planted on the surface of the scaffold or cultured in the scaffold extract had the apparent bone formation and mineralization, and the mineral nodules in the β-TCP/CTS/SBA-15 group were significantly less than the MET/β-TCP/CTS/SBA-15 group. The above information proved that the concentration of MET we selected could promote the osteogenic differentiation of stem cells, which was in line with our purpose.

The ideal scaffold has porosity and pore size characteristics that create an ideal proliferation space for cells and facilitate the exchange of nutrients and metabolite removal. The porosity of the MET/β-TCP/CTS/SBA-15 scaffold was 84.3 ± 4.2%, and the pore size of the scaffold ranged from 100 to 300 μm. These characteristics were consistent with the ideal scaffold requirements [33]. We evaluated the biocompatibility of the scaffold in vitro. SEM observations showed that the surface of the MET/β-TCP/CTS/SBA-15 scaffold was rough, providing a recognition site for cell adhesion, and the BMSCs attached to the scaffold had good morphology and vitality. Through close observation at continuous time points, the results of the CCK-8 experiment indicated that the density and viability of living cells contained in the MET/β-TCP/CTS/SBA-15 group and the β-TCP/CTS/SBA-15 group were not statistically different. The stained images of live and dead cells under the confocal microscope were consistent with the above results, which indicated the excellent biocompatibility of the MET/β-TCP/CTS/SBA-15 scaffold composite scaffold.

The scaffold needs to have osteogenic differentiation ability to promote bone defect repair. Through the previous experiments, we observed that MET/β-TCP/CTS/SBA-15 scaffolds could promote the osteogenic differentiation of BMSCs in vitro. The RT-PCR results indicated that the mRNA expression levels of the bone-related genes Runx2, Col1a1 and BMP-2 in cells within the MET/β-TCP/CTS/SBA-15 scaffolds were significantly higher than those in cells in the β-TCP/CTS/SBA-15 group. The above results indicated that the MET/β-TCP/CTS/SBA-15 group had a better osteogenic performance than the β-TCP/CTS/SBA-15 group, mainly due to the effect of MET. In vivo results were consistent with in vitro results. This new MET/β-TCP/CTS/SBA-15 scaffold simulated the basic structure of bone tissue and promoted the repair of alveolar bone defects in a periodontitis environment. Micro-CT calculation analysis and histological analysis showed that a large amount of new bone was formed in the MET/β-TCP/CTS/SBA-15 scaffold group. The ability to repair alveolar bone defects was significantly higher than that of other groups. Recently, some scholars designed a biodegradable CTS-based MET intrapocket dental film, which effectively inhibited the loss of alveolar bone in rats with periodontitis [34]. From a preventive medicine point of view, their intrapocket dental film focused on preventing the loss of alveolar bone caused by periodontitis, which was primary prevention. The MET/β-TCP/CTS/SBA-15 scaffolds focused on repairing the bone defect in the complex periodontitis environment, which belonged to tertiary prevention. Their experimental results suggested that MET and CTS could inhibit the loss of alveolar bone under periodontitis. The MET/β-TCP/CTS/SBA-15 scaffold containing these two components could further promote alveolar bone defect repair under periodontitis conditions.

This study demonstrated that MET was an effective drug that could promote osteogenic differentiation of BMSCs along osteogenic lineages. MET enhanced the performance of β-TCP/CTS/SBA-15 scaffolds in vitro without the highly cytotoxic effects of chemotherapy drugs. Studies based on different models often produce different results, and further studies are needed to elucidate the mechanism of MET promoting osteogenesis at the molecular level.

## Conclusion

In summary, we successfully developed bifunctional MET/β-TCP/CTS/SBA-15 scaffolds using the freeze-drying method. The scaffolds exhibited a porous structure and a sustained MET release property. In addition, the scaffolds were found to support BMSCs adhesion and promote osteogenesis in vitro. More importantly, we found that the MET/β-TCP/CTS/SBA-15 scaffolds could promote the repair of alveolar bone defects in a periodontitis environment. Our study suggests that the MET-loaded β-TCP/CTS/SBA-15 scaffolds could promote alveolar bone regeneration in a rat model of periodontitis. This new scaffold is a promising candidate for the treatment of alveolar bone defects in a periodontitis environment.
